# The Barnato Legacy

**Published:** 1909-01-09

**Authors:** 


### Editor's Letter-Box.

### THE BARNATO LEGACY.

### A Suggestion with a Protest.

*(From a Correspondent.)*

The munificent bequest left by Mr. Barnato to found a hospital in memory of his brother and nephew throws upon the trustees of the will a very grave responsibility. Such bequests possess inherent potentialities of good and evil, for much harm may be done by misapplied charity. Undoubtedly the trustees will be overwhelmed with numerous proposals, many deserving of thorough consideration. Indeed there has been started already more or less of a campaign in favour of one particular object, namely, the provision of accommodation for the treatment out of London of the tuberculous or the pre-tuberculous child. Even this large legacy would be but a drop in the ocean of gold necessary for the adequate accomplishment of such a scheme, for every child reared in a London slum may be regarded as pre-tuberculous. Not one could be excluded with absolute certainty, seeing that the earliest stages of tuberculosis are often unsuspected, and still more often impossible of certain diagnosis.

The bequest might be devoted to an object which would produce an obvious and direct good or to one which would prove indirectly much more beneficial to mankind universally, though the good effects would not be so immediately apparent. The first desideratum would be realised by the simple erection and endowment of a hospital on the present stereotyped lines. The fructification of the latter could be ensured by devoting the bequest primarily to medical education, and secondarily to hospital treatment.

It is obvious that there are many objections to the establishment of another hospital in London, for there is already a sufficiency thereof, and indeed, in the minds of many, an excessive supply, leading to extravagant competition for patients and charitable contributions. If, however, the trustees decided to devote the money to such a purpose it would be reasonable to take one of the smaller hospitals and enlarge it, providing up-to-date accommodation for the treatment of patients and facilities for the thorough investigation of disease.

One word of warning must be given. It would, for many reasons, be a grave mistake to build a purely sectarian hospital, a Jewish hospital. At first sight this seems an ideal way of carrying out the testator's intentions, but it must on consideration be pronounced unsatisfactory. One of the features of London hospital charities has ever been their unsectarian character. The sick poor, no matter their nationality or their religion, have received every attention in accordance with the nature or the severity of their illness. Even if such a sectarian hospital threw its doors widely open to all comers it would still be looked upon with disfavour. There would be the same need for the provision of separate wards for those of other religious views, as already exists and is provided for in some of our hospitals. There might be a difficulty in obtaining the services of a staff of that particular religion of high enough professional standing, for the medical profession does not seem to appeal very strongly to the Hebrew race as a means of livelihood in this country. Eventually there would be the danger of the public thinking that the rich and charitable members of the chosen race would devote their contributions to the special hospital for their coreligionists, and there would be a tendency to an isolation of this race rather than the fusion, which is more or less going on in the ordinary affairs of life and remains independent of religious views. It would certainly create an ill-will the consequences of which might be far-reaching as well as unforeseen. Already certain hospitals provide special wards, cooks, and kitchens, etc., for those of the Jewish persuasion, and should the trustees be desirous of increasing the accommodation in this direction there is no doubt the bequest would be well expended. But it is from medical education that the greatest good for the greatest number would ultimately be obtained. There is in this country no hospital, on the lines of the Johns Hopkins Hospital at Baltimore, for post-graduate teaching, a hospital founded and maintained by the munificence of private individuals for the treatment of disease and the provision of teaching facilities for qualified medical practitioners. In London it has proved impossible to carry out such teaching efficiently without the hospital beds available for clinical teaching. The London Medical Graduates' College and Polyclinic is contemplating bringing its work to a close on account of the impossibility of obtaining the funds necessary for maintenance.

In spite of a list of over 500 subscribers and ten years' successful teaching work, it has become evident that the public will not provide the sinews of war and that the struggling beginner in the arduous path of professional life has rarely more than enough for his own support. Such funds could be obtained for a hospital carrying on ordinary hospital work in addition to the advanced teaching required by medical graduates. For undergraduate students there is already ample provision.

We commend this suggestion to the careful consideration of the trustees. There are many hospitals with an efficient nucleus of beds, some with ground areas available for building extensions, with an existing organisation, offices, and an excellent honorary staff. All that is needed is the provision of more wards, pathological and other laboratories, libraries, lecture rooms, and the like, for the needs of post-graduate study. It is not essential that such a hospital should be centrally situated, for the means of communication in London are now quite easy and simple. If, in addition, the hospital could be re-christened the "Joel-Barnato" Hospital and special wards and arrangements provided for Jewish patients, the objects of the trust would apparently be efficiently carried out, and in a way which would benefit mankind at large. The indirect benefits derived from post-graduate instruction are enormous, though not immediately obvious. When we look round and wonder at the lowness of the death rate and the diminishing amount of serious illness and try to ascertain the cause, we must admit that it is largely due to improved sanitation, housing, and medical treatment, all of which are the direct or indirect result of the steady progress of medical education and the wide distribution of well-trained members of the medical profession.

